# Structural Features of the Peptide Homologous to 6-25 Fragment of Influenza A PB1 Protein

**DOI:** 10.1155/2013/370832

**Published:** 2013-12-24

**Authors:** Vladimir V. Egorov, Oleg V. Matusevich, Aram A. Shaldzhyan, Alexey N. Skvortsov, Yana A. Zabrodskaya, Yuri P. Garmay, Sergey B. Landa, Dmitry V. Lebedev, Vladimir V. Zarubayev, Alexey K. Sirotkin, Andrey V. Vasin, Oleg I. Kiselev

**Affiliations:** ^1^Department of Molecular Virology, FSBI Research Institute of Influenza, Ministry of Health of the Russian Federation, 15/17, Professor Popova Street, Saint Petersburg 197376, Russia; ^2^Department of Molecular and Radiation Biophysics, Kurchatov Institute, FSBI St. Petersburg Nuclear Physics Institute, Orlova Roscha, Gatchina 188300, Russia; ^3^Faculty of Chemistry, Saint Petersburg State University, Saint Petersburg 198504, Russia

## Abstract

A mirror-symmetry motif was discovered in the N-terminus of the influenza virus PB1 protein. Structure of peptide comprised of the corresponding part of PB1 (amino acid residues 6-25) was investigated by circular dichroism and in silico modeling. We found that peptide PB1 (6-25) in solution assumes beta-hairpin conformation. A truncated peptide PB1 (6-13), containing only half of the mirror-symmetry motif, appeared to stabilize the beta-structure of the original peptide and, at high concentrations, was capable of reacting with peptide to form insoluble aggregates *in vitro*. Ability of PB1 (6-13) peptide to interact with the N-terminal domain of PB1 protein makes it a potential antiviral agent that inhibits PA-PB1 complex formation by affecting PB1 N-terminus structure.

## 1. Introduction

Peptides are a promising basis for the development of drugs. Creating peptide-based drugs includes the search of active sites or functionally important sites in the target protein and the selection of peptides capable of specific interaction with these sites. Such a selection usually can be accomplished using peptide libraries containing random fragments of amino acid sequences (irrational design) or fragments of natural proteins capable of reacting with target structural determinants. Developing approaches to rational design of potential peptide inhibitors of structural functional determinants of proteins can potentially expand the range of therapeutic medications based on peptides [1].

The key role in the process of development of an infection caused by the influenza virus is played by RNA polymerase. RNA polymerase of the influenza virus consists of three subunits—PA, PB1, and PB2. Assembling of PA and PB1 subunits of a polymerase complex occurs in cytoplasm of infected cells. Heterodimer is transported into the nucleus, where, upon accession of the third subunit—PB2, a functional polymerase complex is formed. According to the data of several authors, the critical region of the interaction of proteins PA and PB1 is the N-terminal region of protein PB1 [2, 3]. A number of studies [4, 5] showed that the structural determinant of the PA-PB1 interaction during formation of a polymerase complex of the influenza virus is fragment 1-15 of PB1 protein. The X-ray structural analysis of the crystals of the complex of the fragment of PA protein and the peptides corresponding to N-terminus of PB1 was carried out, and it showed that this fragment forms a system of hydrogen bonds with the C-terminal domain of PA. It was also shown that the presence of the polypeptide, containing the fragment of the amino acid sequence corresponding to amino acid residues 1-25 and 1-15 of PB1, in a cell, prevents the infection of cells with the influenza virus. For example, in [5], genetic construct that expresses protein which contains the given fragment was used to suppress the replication of the influenza virus; in [6], influenza virus replication was blocked by the peptide functionalized with the fragment of human immunodeficiency virus tat protein and such a way transported from the medium to the cell. It was assumed that peptide PB1 (1-25) inside the cell forms a structure close to the structure of the N-terminal region of PB1 in the structure of PA-PB1 complex, interacts with protein PA, and thus prevents the formation of a polymerase complex required for viral replication. This studies show that the structure of the N-terminal region of protein PB1 is critical for the polymerase complex assembly and replication of the influenza virus.

In this work, we investigate the structural features of N-terminal peptide of protein PB1 to design a potential peptide inhibitor of assembly of a polymerase complex of the influenza virus. In contrast to studies [4, 7], where the peptide inhibitor interacted with PA and competed for its site of interaction with PB1, we used the mirror-symmetry motif in the sequence of PB1 to design a peptide that would inhibit PA-PB1 assembly by interaction with the N-terminal part of PB1.

## 2. Materials and Methods

### 2.1. Peptides

Peptides' PB1 (6-13) (TLLFLKVP) and PB1 (6-25) (TLLFLKVPAQNAISTTFPYT) solid phase peptide synthesis was carried out by 2-chlorotrityl chloride resin according to Fmoc-/tBu strategy. For fragments 6-25 protein, PB1 target compound was synthesized from fragments divided by the proline residue at position 8. The completeness of the reactions was evaluated using the Kaiser test for free amino groups. The purification of the compounds obtained was carried out via preparative reversed-phase HPLC. Their purity turned to be not less than 95%; their structure was confirmed by mass-spectrometry HRMS(ESI^+^).

### 2.2. Computer Modeling of the 3-Dimensional Structure of the Peptides

The study of mobility of the peptides was carried out by the method of molecular dynamics (MD) using the software package GROMACS 4.5.5 (http://www.gromacs.org/).

MD calculation of all the systems was performed in cubic cells with applied periodic boundary conditions. The lifetime of the systems was 100 ns. The temperature of the systems was set to 310 K. The integration step was 2 fs. The calculations were performed considering the solvent. The minimum distance from the molecule to the cell wall was 1 nm, which made it possible to avoid the interaction of the peptides in neighboring cells. Energy minimization was performed using the steepest descent algorithm. Cutoff for atom-atom electrostatic and Van der Waals interactions was 1 nm. Long-range electrostatic interactions were calculated by algorithm PME.

### 2.3. Circular Dichroism

Circular dichroism (194–320 nm) was measured on PB1 (6-25) 1 mg/mL solution. Spectra were analyzed with CDNN software.

### 2.4. Peptide Docking

Protein docking was performed using the Hex server software (http://hex.loria.fr/) with default parameters (Van der Waals plus electrostatic mode), the best 10 docking conformations were analyzed.

### 2.5. Transmissive Electron Microscopy

Preparation of samples for electron microscopy was performed according to the standard procedure of negative staining. A drop (20 mkl) of the test sample diluted to the concentration of 0.2 mM was applied on parafilm. A 200-celled copper grid with carbon coating was placed on the drop for 15 s. The base plate was washed with distilled water two times for 15 seconds. The sample was contrasted with 1.5% aqueous solution of sodium salt of phosphotungstic acid (pH 7.4) for 15 seconds, then the sample was dried at room temperature. The study was carried out by electron microscope JEOL JEM 1100 at the accelerating voltage of 80 kV.

### 2.6. Laser Correlation Spectroscopy

Spectra were obtained on laser correlation spectrometer LKS-03 (INTOX, Russia); processing of the spectra was performed using the instrument software. The final concentration of the peptides while measurement was 0.2 mM.

## 3. Results and Discussion

**Figure 1:**
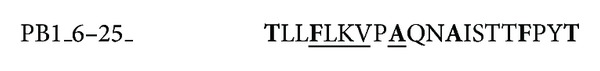
MSM in the sequence of the N-terminal domain of PB1. Amino acid residues in symmetrical positions are printed in bold. Amino acid residues involved in the interaction with PA, according to the data [4, 5], are underlined.

The analysis of the primary structure of the N-terminal domain of PB1 showed that the site of PB1 (6-25) contains a so-called mirror-symmetry motif (MSM) (Figure 1).

The frequency of MSM occurrence near functional sites seems to exceed the average for the amino acid sequences of natural proteins, and it has been suggested that MSM may play a role in the formation of functional sites of proteins, as well as in protein oligomerization [8, 9].

The available X-ray crystallographic structure of PA complex with PB1 N-terminal fragment (amino acids 1–15) shows that, when bound to PA1, the secondary structure of 1–15 N-terminal fragment of PB1 is an alpha-helix [5]. However, at this point, there is no three-dimensional structure of PB1 protein apart from its complex with PA. To investigate the possible structure of the N-terminal domain of the free PB1, we performed computer modeling of the structure and dynamics of PB1 N-terminal fragment.

**Figure 2:**
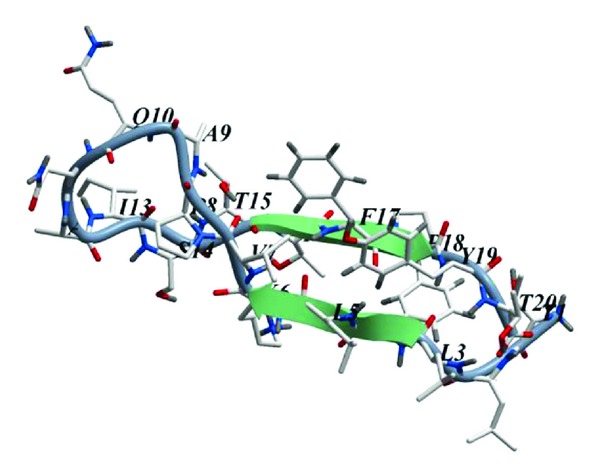
Average 3-dimensional structure of the mirror-symmetric motif 6-25 PB1 according to the data of computer modeling. Beta-strands are represented as arrows. Numbers of residues begin from 1. Visualization using the ICM Molsoft software.

An MSM-containing peptide matching the residues 6-25 of PB1 was chosen for modeling. As a result of the modeling of 3-dimensional structure of the peptide that included 100 ns molecular dynamics simulation in the presence of solvent and by analyzing the trajectories, it was found that the peptide was prone to formation of the a beta-hairpin type structure (Figure 2). The peptide acquired this conformation in the early stages of the simulation, while further simulation resulted only in minor alterations that did not affect the secondary structure of the peptide. Backbone H-bonds L9-Y24 and L11-F22 (PB1 numbering) were crucial for stabilizing the beta-hairpin conformation.

**Figure 3:**
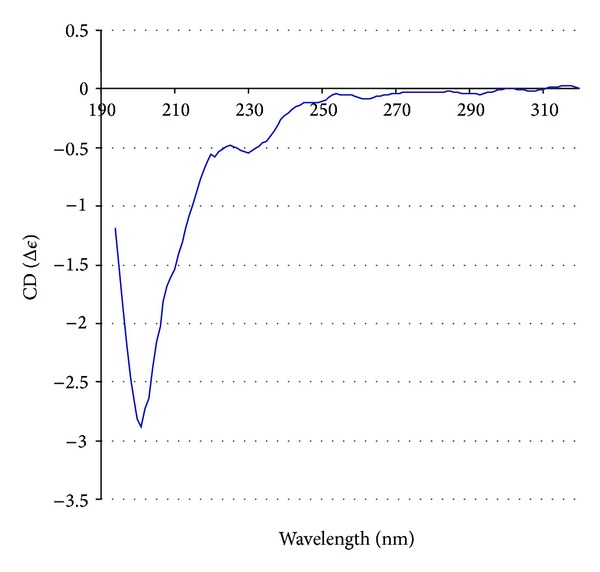
Circular dichroism spectrum of peptide PB1 (6-25).

The study of the secondary structure using circular dichroism (CD) (Figure 3) revealed that the peptide PB1 (6-25) in solution has a structure similar to the described model of beta-hairpin obtained using molecular dynamics methods (8% of alpha-helix, 30% of antiparallel beta-sheet, 25% of beta-turns, and 37% of random coil structure). The beta-type structures appear to, therefore, prevail in the PB1 (6-25) peptide and may represent the native conformation of PB1 N-terminus, while the formation of the alpha-helix observed by X-ray crystallography could likely be the result of PB1 interaction with the polymerase subunit.

The area crucially important for PB1 interaction with PA (FLKV, 4–7 residues), according to the results of the modeling, was located in a beta-sheet, in contrast to the data presented in [5], where these residues were located in an alpha-helix. One could suppose that stabilization of the beta-structured state of PB1 N-terminus would result in inhibition of the interaction of this protein with PA, polymerase complex assembly, and reproduction of viral RNA. A peptide which would stabilize the beta-hairpin structure of PB1 (6-25) peptide is, therefore, a good candidate for PB1-PA interaction inhibitor.

**Figure 4:**
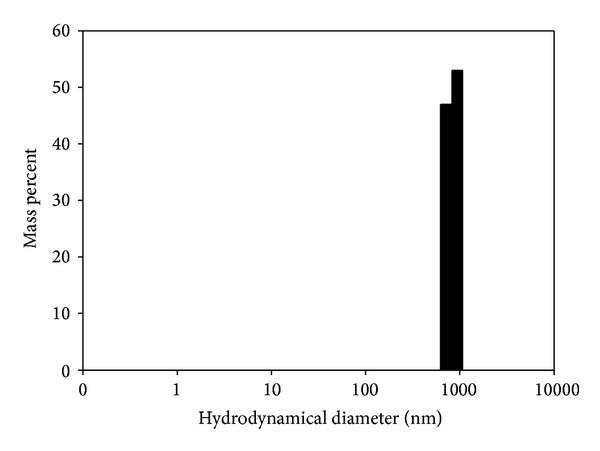
Results of laser correlation spectroscopy of the aggregates formed in the solution as the result of the interaction of peptides PB1 (6-25) and PB1 (6-13).

Considering the MSM in PB1 (6-25), we investigated its interactions with PB1 (6-13) peptide that was a half the original peptide. When mixing the solutions of peptides PB1 (6-13) and PB1 (6-25) at high concentrations (0.5 mM and higher) and incubating the solution at room temperature for an hour, turbidity was observed in the sample containing the mixture. The study of the diluted solution by laser correlation spectroscopy showed that aggregates with the diameter of 500–1000 nm (Figure 4) are formed.

**Figure 5:**
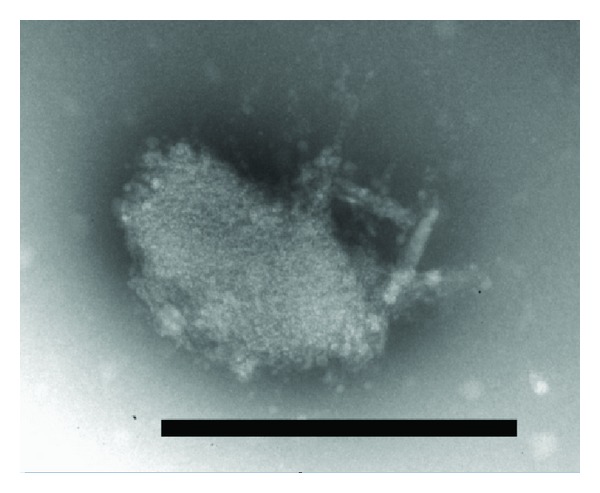
Electron microscopy of a typical aggregate formed in the mixture of peptides PB1 (6-13) and PB1 (6-25). The length of the black rectangle is 500 nm.

The study of peptides PB1 (6-25) and PB1 (6-13) and their mixture by electron microscopy confirmed that structured aggregates with the characteristic linear size of about 500 nm were formed in the mixture (Figure 5), but not in the samples containing either one of the peptides. Thus, the ability of peptides PB1 (6-13) and PB1 (6-25) to interact was demonstrated *in vitro*. It should be noticed that the unsoluble aggregates formed could not reverse into soluble forms after dilution or heating.

**Figure 6:**
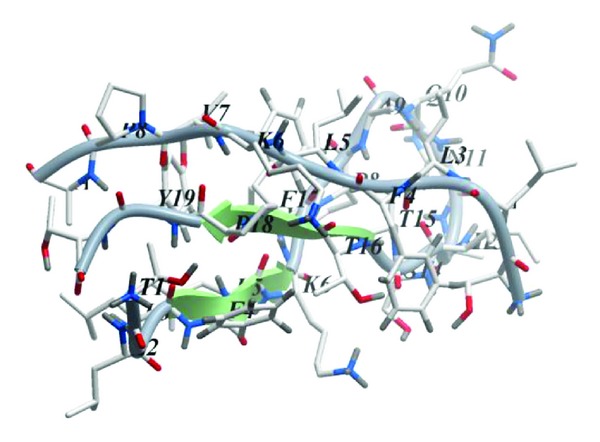
Docking model of the mirror-symmetric motif PB1 (6-25) interacting with PB1 (6-13). Beta-strands are represented as arrows. Numbers of residues begin from 1. Visualization using the ICM Molsoft software.

The modeling of the 3-dimensional structure of PB1 (6-13) peptide using the methods of molecular dynamics showed that the peptide has linear structure; that is, it is not flexible and does not form intramolecular bonds. Using protein docking, we make a model of PB1 (6-25)–PB1 (6-13) interaction (Figure 6). Analysis of the model contact map showed that PB1 (6-13) binds to 14–25 residues' fragment of PB1 (6-25) and forms 2 backbone H-bonds, indicating the possible stabilization of PB1 (6-25) beta-hairpin structure by this peptide.

**Figure 7:**
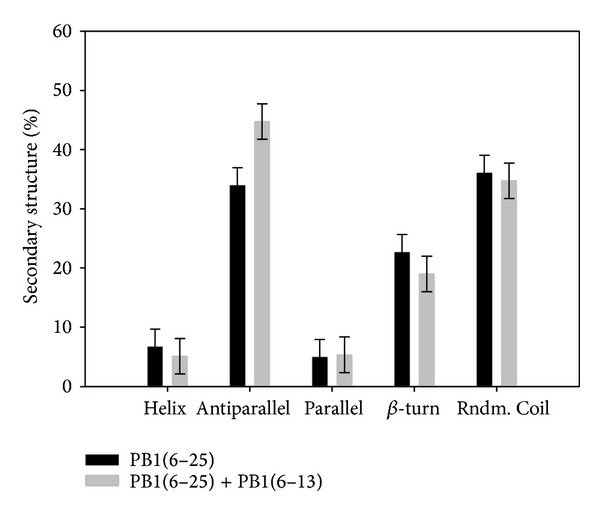
PB1 (6-13) influence on the secondary structure of PB1 (6-25). Results of the differential spectrum analysis with CDNN software.

CD spectroscopy study of PB1 (6-13) peptide influence on the secondary structure of PB1 (6-25) showed that, in equimolar ratio on concentrations were near critical (0.5 mM) PB1 (6-13) contributed to increase antiparallel beta-sheets in latter peptide. The results of differential spectrum analysis were showed on the Figure 7. We use CD spectroscopy at concentrations which were significantly lower than critical to estimate the dissociation constant *K*_*d*_ for PB1 (6-25)–PB1 (6-13) interactions. The concentrations in the mixtures were calculated using bimolecular reversible binding model with one parameter (*K*_*d*_). The CD spectra of the mixtures were then modelled using the calculated concentrations by multidimensional linear square fit. The spectra of pure peptides were also added to data matrix with respective concentration fixed to known values. Total RMSE of the model was then calculated, and the dependency of RMSE on *K*_*d*_ was plotted. The spectral data were found to be close to degenerate (small difference of the spectrum of the complex from the linear combination of the components' spectra) but the model gives a lower bound for *K*_*d*_, which is located approximately in the range of 0.2–1 mM.

The known affinity of PA to PB1 (1-15) (*K*_*d*_~1.6 *μ*M) [4] is much higher than these values. Apart from competitive inhibition of PA binding, one can consider other possible mechanisms of PB1 (6-13) action on PB1 protein such as catalysing the change in the secondary structure of PB1 (6-25) from alpha-helix to beta-sheet, or, at higher concentrations, stimulation of unsoluble aggregates' formation. Stabilizing of PB1 N-terminus beta-sheet secondary structure could make it sterically incompartible to PA binding cave and inhibit PBA-PB1 complex de novo formation.

## 4. Conclusion

We have shown that the mirror-symmetry motif found in the N-terminal region of PB1 protein, which is known to be a part of an alpha-helix in PA-PB1 complex, can likely exist in the beta-conformation in the free PB1 subunit. Peptide PB1 (6-13), containing half of the mirror-symmetry motif, can interact with PB1 (6-25), possibly stabilizing its beta-conformation, that at high concentration, leads to formation of insoluble aggregates *in vitro*. Such an ability could make PB1 (6-13) a basis for a potential antiviral agent that would inhibit PB1-PA complex formation by affecting PB1 N-terminus structure.

## References

[1] Vlieghe P, Lisowski V, Martinez J, Khrestchatisky M (2010). Synthetic therapeutic peptides: science and market. *Drug Discovery Today*.

[2] Torreira E, Schoehn G, Fernández Y (2007). Three-dimensional model for the isolated recombinant influenza virus polymerase heterotrimer. *Nucleic Acids Research*.

[3] Perez DR, Donis RO (2001). Functional analysis of PA binding by influenza A virus PB1: effects on polymerase activity and viral infectivity. *Journal of Virology*.

[4] Wunderlich K, Juozapaitis M, Ranadheera C (2011). Identification of high-affinity PB1-derived peptides with enhanced affinity to the PA protein of influenza A virus polymerase. *Antimicrobial Agents and Chemotherapy*.

[5] He X, Zhou J, Bartlam M (2008). Crystal structure of the polymerase PAC-PB1N complex from an avian influenza H5N1 virus. *Nature*.

[6] Obayashi E, Yoshida H, Kawai F (2008). The structural basis for an essential subunit interaction in influenza virus RNA polymerase. *Nature*.

[7] Ghanem A, Mayer D, Chase G (2007). Peptide-mediated interference with influenza A virus polymerase. *Journal of Virology*.

[8] Shpakov AO (2000). The mirror-type internal symmetry in the primary structure of proteins: detection and functional role. (Review). *Journal of Evolutionary Biochemistry and Physiology*.

[9] Egorov VV, Garmaj YP, Solovyov KV (2007). Amyloidogenic peptide homologous to *β*-domain region of *α*-lactalbumin. *Doklady Biochemistry and Biophysics*.

